# Inactivation of hepatic ATRX in *Atrx* Foxg1cre mice prevents reversal of aging-like phenotypes by thyroxine

**DOI:** 10.18632/aging.101462

**Published:** 2018-06-07

**Authors:** Megan E. Rowland, Yan Jiang, Frank Beier, Nathalie G. Bérubé

**Affiliations:** 1 Departments of Paediatrics and Biochemistry, Schulich School of Medicine and Dentistry, Western University, London, ON, Canada; 2 Children’s Health Research Institute, London, ON, Canada; 3 Department of Physiology and Pharmacology, Schulich School of Medicine and Dentistry, Western University, London, ON, Canada; 4 Western Bone and Joint Institute, Western University, London, ON, Canada

**Keywords:** ATRX, premature aging, thyroid hormone, IGF-1, transcription

## Abstract

ATRX is an ATP-dependent chromatin remodeler required for the maintenance of genomic integrity. We previously reported that conditional *Atrx* ablation in the mouse embryonic forebrain and anterior pituitary using the Foxg1cre driver causes reduced health and lifespan. In these mice, premature aging-like phenotypes were accompanied by low circulating levels of insulin-like growth factor 1 (IGF-1) and thyroxine (T4), hormones that maintain stem cell pools and normal metabolic profiles, respectively. Based on emerging evidence that T4 stimulates expression of IGF-1 in pre-pubertal mice, we tested whether T4 supplementation in *Atrx* Foxg1cre mice could restore IGF-1 levels and ameliorate premature aging-like phenotypes. Despite restoration of normal serum T4 levels, we did not observe improvements in circulating IGF-1. In the liver, thyroid hormone target genes were differentially affected upon T4 treatment, with *Igf1* and several other thyroid hormone responsive genes failing to recover normal expression levels. These findings hinted at Cre-mediated *Atrx* inactivation in the liver of *Atrx* Foxg1cre mice, which we confirmed. We conclude that the phenotypes observed in the *Atrx* Foxg1cre mice can be explained in part by a role of ATRX in the liver to promote T4-mediated *Igf1* expression, thus explaining the inefficacy of T4 therapy observed in this study.

## Introduction

The α-Thalassemia/mental retardation X-linked (ATRX) chromatin remodeler is a component of constitutive heterochromatin [1-5] and is exclusively nuclear [4]. The C-terminus of ATRX contains a Switch/Sucrose non-fermenting (Swi/Snf) domain [6] which confers the ATPase and translocase activity of ATRX necessary for nucleosome remodelling [7]. The N-terminus of ATRX contains an ADD domain (ATRX-DNTM3-DNTM3L) with homology to the

DNA methyltransferase family and is capable of binding methylated histone tails within chromatin [8].

ATRX is essential for heterochromatin [5,9] and telomere integrity [9,10], as well as normal gene expression [11-15]. Deletion of ATRX in neuroprogenitor cells (NPCs) causes DNA damage at telomeres and pericentromeric heterochromatin [10]. We have previously shown that telomestatin-mediated stabilization of G-quadruplexes exacerbated DNA damage and decreased viability of NPCs in the absence of ATRX [10]. ATRX may be facilitating the progression of DNA replication and transcriptional machinery through these non-canonical DNA structures [10,12].

We previously reported that postnatal health and longevity are severely affected in mice with forebrain and anterior pituitary-specific deficiency for ATRX (*Atrx*_Foxg1cre) [10]. Many of the abnormalities detected in these mice resemble those previously reported in mouse models of progeria, including low circulating levels of IGF-1 [16-20]. Progeroid syndromes are a group of disorders characterized by accelerated physiological aging. These heritable disorders produce signs of premature aging in many, but not all tissues and therefore are termed segmental progerias. It has been postulated that in progeroid syndromes, mutations in genome repair genes result in increased genomic and heterochromatin instability [21,22]. This is paired with the downregulation of genes involved in growth, such as *Igf1*, presumably in order to survive the damage [23]. However, if this occurs during development, it is detrimental to the organism, resulting in metabolic defects and premature aging. Several progeroid syndromes have been described, such as Cockayne syndrome [24], Werner syndrome [25] and Hutchinson-Gilford progeria [26]. Symptoms of these disorders typically include reduced growth, decreased lifespan, bone and organ abnormalities and loss of subcutaneous fat.

The role of insulin signalling in aging and longevity is highly conserved [27]. IGF-1 is predominantly produced by the liver and important for stem cell proliferation in many organs and some evidence points to depleted stem cell pools as a main cause of aging [28,29]. A case in point is Hutchinson-Gilford progeria, which is caused by mutations in Zmpste24, a metallopeptidase involved in the processing of lamin A [19]. When lamin A is mutated, the nuclear membrane is unable to form properly, resulting in DNA damage and genome instability [19]. Zmpste24 null mice exhibit accelerated aging phenotypes and low levels of circulating IGF-1 [19]. Remarkably, treatment of these mice with IGF-1 results in amelioration of aging features and extends lifespan, demonstrating that reduction of IGF-1 levels is a cause rather than an effect of premature aging [19].

Xing et al. (2012) showed that mice deficient in thyroid hormone also exhibit premature aging phenotypes associated with greater than 50% decrease in *Igf1* expression in liver and bone [30]. In this case, treatment with both the prohormone thyroxine (T4) and the active hormone triiodothyronine (T3) rescued IGF-1 levels as well as the phenotypic defects [30]. Thyroid hormone mediated gene expression is regulated through the binding of T3 to the thyroid hormone receptors (Thrs). Thrs act as transcription factors to either enhance or repress transcription depending on whether T3 is bound or not [31,32]. Moreover, Visser et al. (2016) reported suppressed thyroid hormone signaling and increased expression of the thyroid hormone deactivating enzyme deiodinase 3 (Dio 3) in mouse models of Xeroderma pigmentosa, Cockayne syndrome and in wild type aged mice [33]. Taken together, this evidence suggests that thyroid hormone signalling is implicated in progeria through the regulation of IGF-1 in early postnatal development.

Given that the *Atrx* Foxg1cre mice exhibit signs of accelerated aging as well as reduced levels of T4 and IGF-1, we hypothesized that T4 treatment could rescue abnormalities in these mice and perhaps extend longevity. Our findings demonstrate that, contrary to our expectation, T4 administration does not rescue IGF-1 serum levels nor the associated adverse phenotypes. Upon further investigation, we found that *Atrx* is partially deleted in the liver of *Atrx* Foxg1Cre mice and is required for the expression of a subset of thyroid hormone responsive genes, including *Igf1*, providing a potential explanation for the lack of rescue in this model system.

## RESULTS

### T4 administration does not improve body growth of *Atrx* Foxg1cre mice

**Figure 1:**
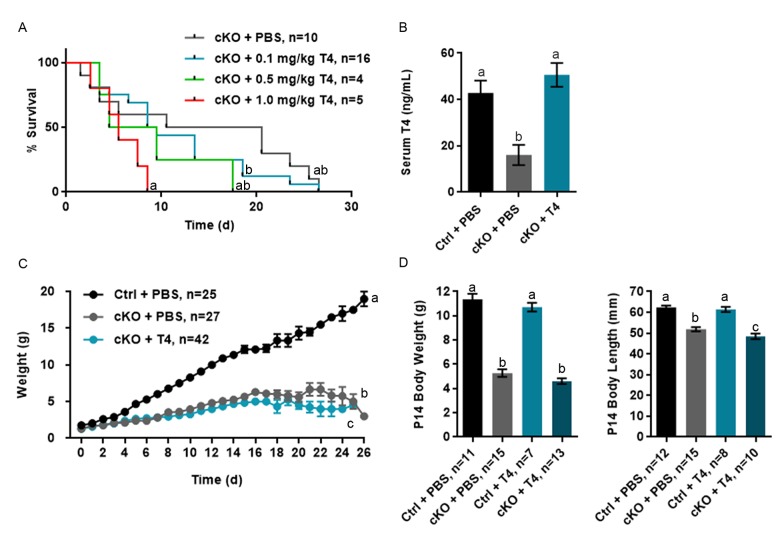
**T4 administration restores normal serum T4 levels in *Atrx* Foxg1cre (cKO) mice but does not rescue life span or growth abnormalities.** (**a**) Kaplan-Meier curve depicting survival of *Atrx* Foxg1cre mice injected with various amount of T4 from P0-P14 (**b**) Serum T4 levels are restored in P14 *Atrx* Foxg1cre mice after T4 treatment (0.1 mg/kg T4). T4 treatment does not improve body size (**c**) or body weight and length (**d**) in *Atrx* Foxg1cre at P14. Groups with the same letter have means that are not significantly different. Groups with different letters have means that are significantly different (*p*<0.05). Error bars represent SEM.

We previously reported that *Atrx* Foxg1cre mice have reduced weight, length and a very short lifespan associated with low T4 and IGF-1 [10]. To test whether T4 injections in the postnatal period might ameliorate deleterious outcomes in these mice, we established a protocol consisting of daily injections of three different doses of L-thyroxine (T4) or PBS as a control, based on Xing et al. 2012 [30]. Cohorts of *Atrx* Foxg1cre (indicated as cKO in figures) and control mice (indicated as Ctrl in figures) were injected subcutaneously with 0.1, 0.5 or 1.0 mg/kg T4 daily from birth to P14. *Atrx* Foxg1cre mice injected with T4 did not show lifespan extension at any dose of T4 (Figure 1A). Furthermore, injection protocols with 0.5 and 1.0 mg/kg resulted in decreased average lifespan compared to *Atrx* Foxg1cre mice + PBS (Figure 1A and Supplementary Figure 1). We thus used the lowest dose of T4 (0.01 mg/kg) for subsequent analyses. We confirmed that at this lower dose, T4 levels were restored to control levels and were significantly increased compared to sham-treated *Atrx* Foxg1cre mice (Figure 1B). If T4 levels in *Atrx* Foxg1cre mice differed from the control mean by more than 2 standard deviations, they were considered biological outliers and removed from the study (Supplementary Figure 2A). In order to determine whether serum T3 (the active form of thyroid hormone) was increased after T4 treatment in *Atrx* Foxg1cre mice, a T3 ELISA was performed (Supplementary Figure 2B). However, due to the low levels of T3 in the serum (1 ng/mL) we were unable to detect a change in any of the groups at this low level.

As previously reported, we observed that the weight of PBS-injected *Atrx* Foxg1cre mice was significantly lower than PBS-injected control mice over time and this effect was not rescued following T4 treatment (Figure 1C). Weight and length measurements at P14 show that both PBS- or T4-injected *Atrx* Foxg1cre mice weigh less than half of controls and are shorter in length (Figure 1D). These results indicate that, contrary to our expectation, restoration of normal circulating T4 levels in *Atrx* Foxg1cre mice failed to improve their reduced size and short lifespan.

### Low subcutaneous fat and blood glucose are not improved by T4 administration

**Figure 2:**
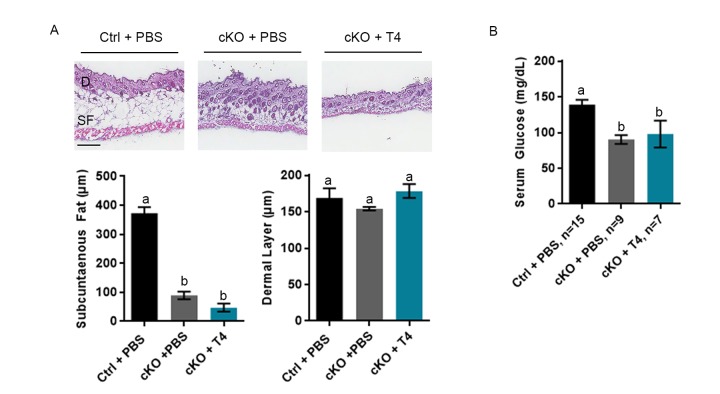
**Low subcutaneous fat or serum glucose are not rescued by T4 administration in *Atrx* Foxg1cre (cKO) mice.** (**a**) H & E staining of P20 skin cryosections shows that subcutaneous fat (SF) is not restored to control levels in *Atrx* Foxg1cre mice following T4 treatment (n=3). Dermal layer (**D**) was unchanged between control and either PBS or T4 treated *Atrx* Foxg1cre mice as previously reported (Scale bar = 200 µm). (**b**) Serum glucose at P14 was not restored to control levels in *Atrx* Foxg1cre mice following T4 treatment. Groups with the same letter have means that are not significantly different. Groups with different letters have means that are significantly different (*p*<0.05). Error bars represent SEM.

The *Atrx* Foxg1cre mice were previously reported to have very low levels of subcutaneous fat and serum glucose [10]. To measure the effects of T4 injections on these parameters, we examined skin sections and blood taken from P20 *Atrx* Foxg1cre mice treated with PBS or T4. These analyses confirmed our previous findings that subcutaneous fat thickness was significantly reduced in *Atrx* Foxg1cre mice compared to controls; however, it was not improved in T4-treated *Atrx* Foxg1cre mice and appears to be further reduced (Figure 2A). In contrast, thickness of the dermal layer was not affected by the deletion of *Atrx* or T4 treatment. A similar outcome was observed with serum glucose level, which was significantly decreased in both PBS- and T4-treated *Atrx* Foxg1cre mice compared to control mice (Figure 2B). Additionally, the liver and spleen of PBS-treated *Atrx* Foxg1cre mice were decreased in size when compared to control. Upon T4 treatment, we observed a slight increase in liver and spleen weights (relative to body weight) in *Atrx* Foxg1cre mice, but it was not significantly different from either PBS treated or control mice (Supplementary Figure 3). At P20, heart weight was unchanged in *Atrx* Foxg1cre treated with PBS compared to control, however following treatment with T4, heart weight increased significantly (Supplementary Figure 3). In summary, subcutaneous fat and serum glucose levels were not rescued by T4 treatment in *Atrx* Foxg1cre mice.

### T4 administration fails to recover circulating and hepatic IGF-1 levels

**Figure 3:**
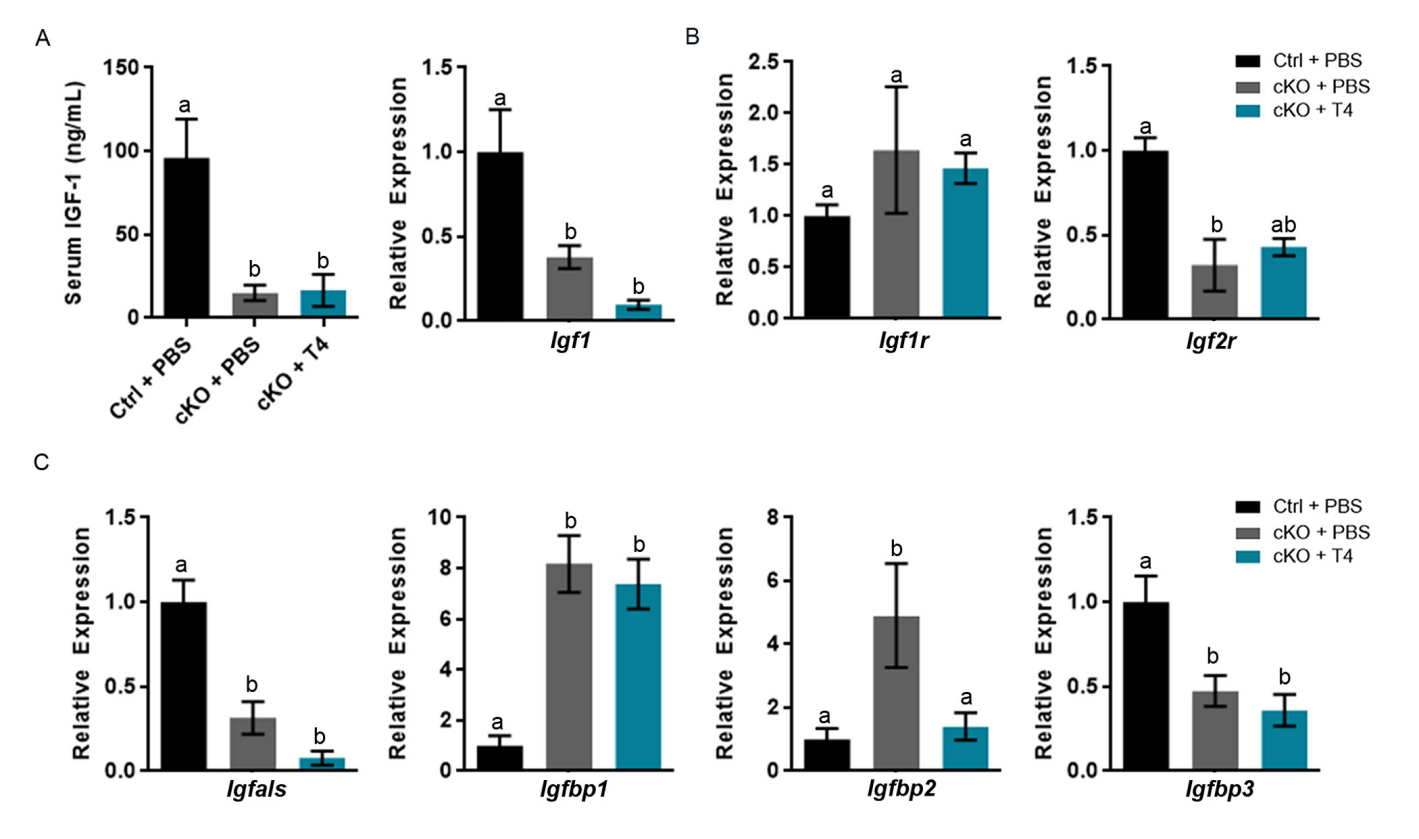
**Altered gene expression and serum IGF-1 levels are not ameliorated by T4 administration (cKO) mice at P14.** (**a**) Serum IGF-1 and expression of liver *Igf1* are not restored to control levels following T4 treatment in *Atrx* Foxg1cre mice. (**b**) qRT-PCR of *Igfr* and *Igf2r* expression following T4 treatment in *Atrx* Foxg1cre mice. (**c**) *Igfals,* *Igfbp1, Igfbp2 and Igfbp3* (IGF-1 complex proteins) transcript levels in the liver upon T4 treatment. qRT-PCR was normalized to β-actin expression, n=3-6. Groups with the same letter have means that are not significantly different. Groups with different letters have means that are significantly different (*p*<0.05). Error bars represent SEM.

We confirmed that *Atrx* Foxg1cre serum IGF-1 level at P14 is 17.7% that of control mice (Figure 3A). However, recovery of normal T4 circulating levels did not result in the expected increase of serum IGF-1. Given that the liver is the major source of circulating IGF-1, we next examined liver *Igf1* expression by quantitative reverse transcriptase PCR (RT-qPCR) and found that *Igf1* expression is significantly decreased in the liver of *Atrx* Foxg1cre mice, even after T4 supplementation (Figure 3A).

The decrease in serum IGF-1 in *Atrx* Foxg1cre mice could be due to alterations in *Igf1* receptor (*Igf1r)* or *Igf2* receptor (*Igf2r*) expression. IGF-1 binds Igf1r*,* which elicits an intracellular response, whereas Igf2r acts as a scavenger receptor and no response occurs. While *Igf1r* expression is not altered in the liver, *Igf2r* expression is decreased in the *Atrx* Foxg1cre compared to control as previously reported [10] and not recovered by T4 treatment (Figure 3B). These data provide evidence that IGF-1 is able to bind Igf1r and is not subject to deactivation by high levels of Igf2r.

Serum IGF-1 could also be decreased due to gene expression alterations in IGF binding proteins (IGFBPs) and acid labile subunit (IGFALS). Both IGFALS and IGFBPs bind IGF-1, enhancing or diminishing IGF-1 signaling depending on the need of the cell. *Igfals* and *Igfbps* are aberrantly expressed in livers of *Atrx* Foxg1cre mice compared to controls*. Igfals* expression is significantly decreased in *Atrx* Foxg1cre mice + PBS compared to control and is not rescued by T4 treatment. *Igfbp1* and *Igfbp2* expression are significantly increased in *Atrx* Foxg1cre compared to control mice. *Igfbp2*, but not *Igfbp1* expression is restored to control levels in *Atrx* Foxg1cre + T4. Low *Igfbp2* expression has been reported in hypothyroidism and its increase in expression is likely due to a feedback mechanism following increased serum T4. *Igfbp3* expression is decreased in the *Atrx* Foxg1cre + PBS and not restored to control levels following T4 treatment (Figure 3C) These results indicate that despite restoring serum T4 levels, most genes involved in IGF-1 signalling are still aberrantly expressed.

### T4 administration restores thyroxine binding globulin gene expression and a subset of thyroid hormone responsive genes in the liver of *Atrx* Foxg1cre mice

**Figure 4:**
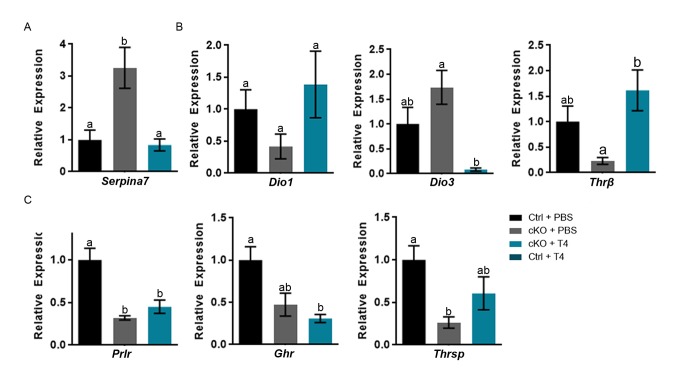
**T4 administration restores thyroxine binding globulin gene expression and a subset of thyroid hormone responsive genes in the liver of *Atrx* Foxg1cre (cKO) mice at P14.** (**a**) *Serpina7* (thyroxine binding globulin) gene expression is restored following T4 treatment. (**b**) Administration of T4 restores thyroid hormone responsive gene expression of *Dio1* and *Thrβ* in *Atrx* Foxg1cre mice to that of control mice. In addition, there is a dramatic repression of *Dio3* expression in the liver of *Atrx* Foxg1cre mice at P14. (**c**) Despite normal expression of *Thrβ*, some thyroid hormone responsive genes are not rescued following T4 treatment in *Atrx* Foxg1cre mice. qRT-PCR data were normalized to β-actin expression, n=3-6. Groups with the same letter have means that are not significantly different. Groups with different letters have means that are significantly different (*p*<0.05). Error bars represent SEM.

Alterations in the gene *Serpina7* (thyroxine binding globulin (TBG)), one of the thyroid hormone transport molecules, could also affect transport of T4. *Serpina7* is significantly increased in the *Atrx* Foxg1cre + PBS and is restored to control levels following treatment with T4 (Figure 4A). We next examined the effect of T4 injections on the expression of several thyroid hormone target genes in the liver of *Atrx* Foxg1cre mice. *Dio1*, which converts T4 to its active form T3, is expressed at slightly decreased levels in *Atrx* Foxg1cre liver, however not significantly. Dio3, the enzyme which converts T3 to the inactive forms rT3 and T2, is normally expressed embryonically when precise control over thyroid hormone signalling is crucial. *Dio3* was reported to be reactivated in the liver of two mouse models of premature aging and in normal mouse aging [33]. *Dio3* is slightly increased in *Atrx* Foxg1cre mice compared to control, however not significantly (Figure 4B). Following T4 treatment *Dio3* expression is highly repressed and is significantly decreased in *Atrx* Foxg1cre mice + T4 compared to *Atrx* Foxg1cre mice + PBS, suggesting that T4 is converted to the T3 active form. *Thyroid hormone receptor β* (*Thrβ*), the active thyroid hormone receptor in the liver, is decreased in the *Atrx* Foxg1cre + PBS compared to control. When treated with T4, *Thrβ* expression is restored to control levels and is significantly increased compared to *Atrx* Foxg1cre + PBS (Figure 4B).

Despite proper regulation and activation of thyroid hormone in the liver, expression of thyroid hormone responsive genes *Prlr*, *Ghr* and *Thrsp* were not restored to control levels in *Atrx* Foxg1cre mice treated with T4 (Figure 4C). While the liver of *Atrx* Foxg1cre mice should essentially be normal, all these results suggested that this is not the case and that it fails to respond to normal thyroid hormone signalling. This prompted us to investigate the possibility that Foxg1Cre expression might be spuriously activated in the liver of these mice, causing *Atrx* gene inactivation.

### Cre recombinase expression and *Atrx* deletion in the liver of *Atrx* Foxg1cre mice

**Figure 5:**
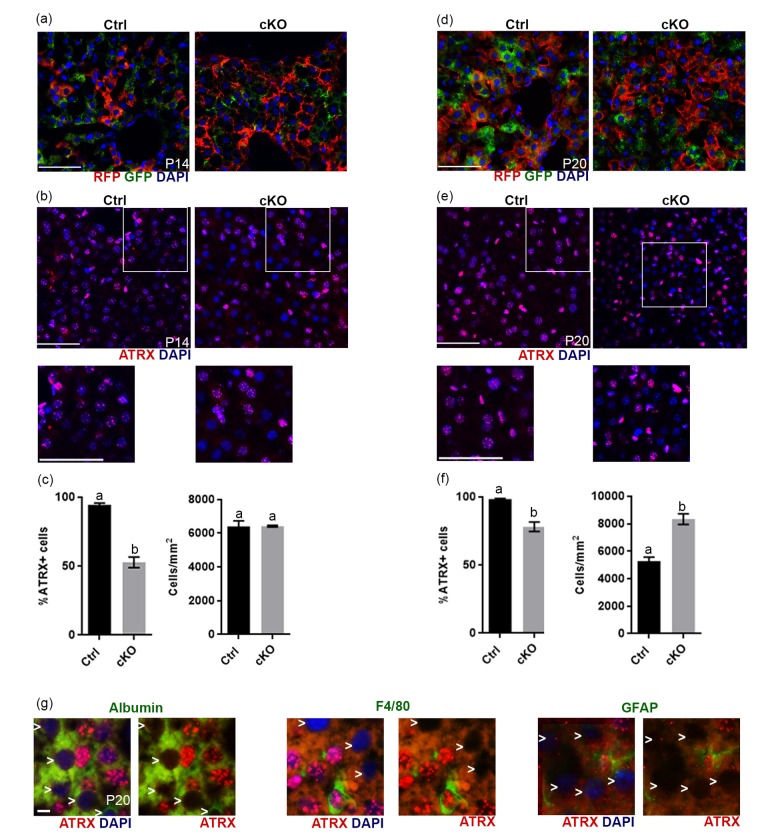
**Evidence of Cre recombinase expression and *Atrx* deletion in a subset of hepatocytes in *Atrx* Foxg1cre (cKO) mice at P14 and P20.** (**a**) Immunofluorescence detection of RFP and GFP in *Atrx* Foxg1cre;ROSAmT/mG mice reveals Cre-mediated GFP expression (**b**) ATRX immunofluorescence of liver cryosections at P14 shows that many nuclei do not express ATRX protein. White box outlines magnified area below. Scale bar = 50 µm. Representative image of n=3 Ctrl/cKO pairs. (**c**) Cell counts of ATRX+ cells reveal a significant reduction in the proportion of ATRX+ cells in *Atrx* Foxg1cre compared to control liver at P14 despite equal number of cells. (**d**) Immunofluorescence detection of RFP and GFP in P20 liver of *Atrx* Foxg1cre; ROSAmT/mG mice shows expression of shows Cre-mediated GFP expression. (**e**) ATRX staining in liver cryosections at P20 shows presence of ATRX-null nuclei and the accumulation of bright ATRX+ cells. White box outlines magnified area below. Scale bar = 50 µm. (Representative images from n=3 Ctrl/cKO pairs). (**f**) Cell counts show a significant reduction in the proportion of ATRX+ cells and increased total cell density in *Atrx* Foxg1cre liver at P20 compared to controls (**g**) Immunofluorescence staining of P20 *Atrx* Foxg1cre liver shows that cells lacking ATRX co-stain with albumin (hepatocytes) but not with F4/80 (Kupffer cells) or GFAP (stellate cells). White arrows point to ATRX-null nuclei. Scale bar = 5 µm. Original magnification, 40x. In graphs (**c**) and (**f**), groups with the same letter have means that are not significantly different and groups with different letters have means that are significantly different (*p*<0.05). Error bars represent SEM.

We had previously verified by western blot analysis that ATRX protein levels were normal in the liver in P20 *Atrx* cKO mice [10]. However, given the results above, we re-examined the status of ATRX in the liver. To achieve this, we introduced the ROSAmT/mG reporter allele into the *Atrx* Foxg1cre mice to allow immunofluorescent detection of Cre-recombinase activity through the detection of green fluorescent protein (GFP), while cells that have never been exposed to Cre-recombinase activity display red fluorescent protein (RFP). Immunofluorescence detection of RFP and GFP in liver cryosections at either P14 (Figure 5A) or P20 (Figure 5D) in *Atrx^wt/Y^; Foxg1cre; Rosa mTmG* (controls) and *Atrx^f/Y^;Foxg1cre;Rosa mTmG* (cKO) mice revealed the presence of many GFP+ cells in the liver. To verify that this resulted in loss of ATRX protein, we performed immunofluorescence staining of ATRX in liver cryosections at either P14 (Figure 5B) or P20 (Figure 5E). Quantification revealed a significant reduction in the proportion of cells that express ATRX in the liver of *Atrx* Foxg1cre mice compared to controls at both P14 (Figure 5C) and P20 (Figure 5F). These results indicate that Cre-mediated *Atrx* inactivation occurred in the liver of *Atrx* Foxg1cre mice. However, at P20, we noted an increase in the total number of cells in the *Atrx* Foxg1cre liver, and that many oblong-shaped cells expressed high levels of ATRX when compared to control (Figure 5F). We speculate that these cells represent a regenerative event mediated by proliferating ATRX+ Kupffer or stellate cells, likely explaining the lack of difference in the levels of ATRX protein previously observed in the P20 liver of *Atrx* Foxg1cre mice by Western blot analysis [10]. Immunofluorescence detection of ATRX and either albumin (hepatocyte marker), F4/80 (Kupffer cell marker) or GFAP (stellate cell marker) in *Atrx* Foxg1cre mice revealed that cells lacking ATRX co-express albumin but not F4/80 or GFAP, indicating that Cre-mediated *Atrx* deletion occurs in hepatocytes (Figure 5G).

### Increased expression of subset of thyroid hormone responsive genes upon T4 administration require ATRX expression

**Figure 6:**
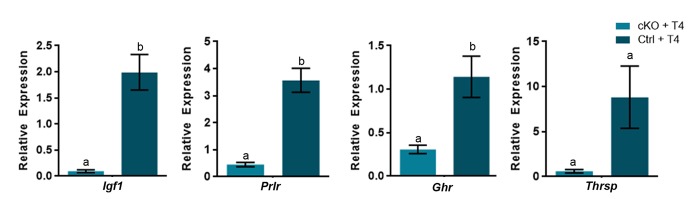
**A subset of thyroid hormone responsive genes are induced in the P14 liver in the presence of excess T4, in an ATRX-dependent manner.** Administration of T4 induces gene expression at or above the level of controls treated wtih PBS (control set to 1) only when ATRX is present in Ctrl + T4 mice and not in *Atrx* FoxG1cre (cKO) + T4 mice at P14. qRT-PCR data were normalized to β-actin expression, n=3-6. Groups with the same letter have means that are not significantly different. Groups with different letters have means that are significantly different (*p*<0.05). Error bars represent SEM.

**Figure 7:**
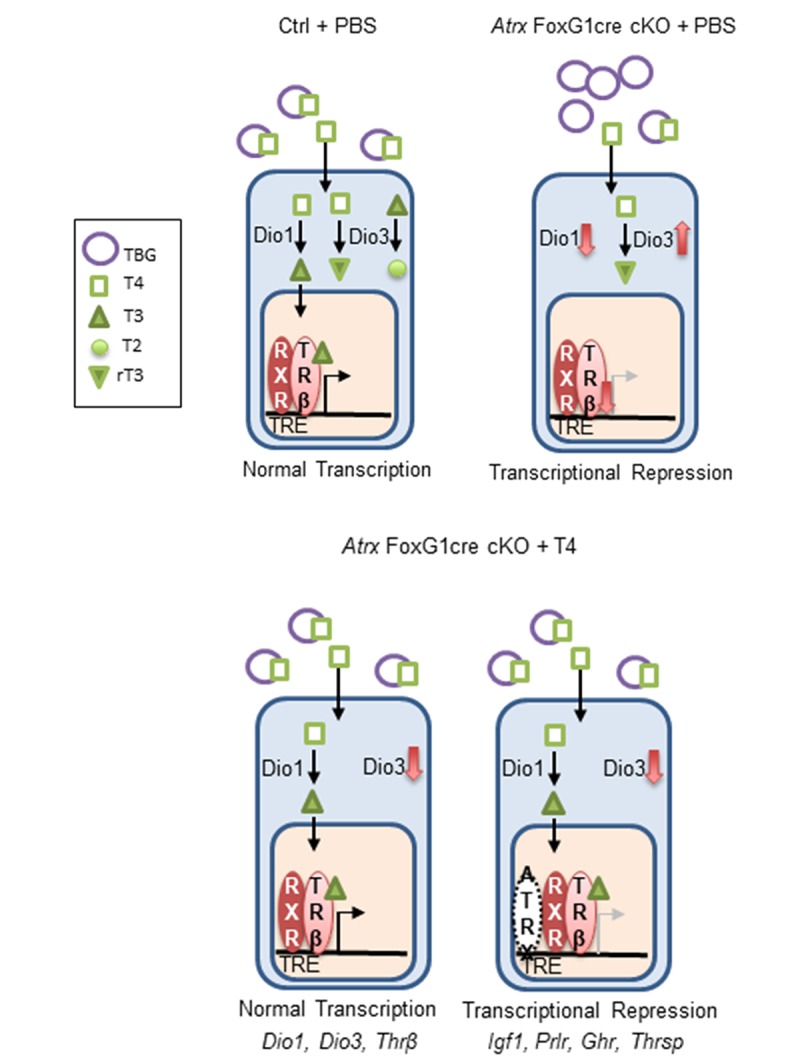
**ATRX is required for the transcription of several thyroid hormone responsive genes in the liver.** In the control, T4 is bound to thyroxine binding globulin (TBG) in the blood. It is converted to T3 in the liver by *Dio1*. Both T4 and T3 can be inactivated by *Dio3* to produce the inactive molecules T2 and rT3. T3 binds its receptor *Thrβ* (found as a heterodimer with retinoid x receptor), which enters the nucleus, binds a thyroid hormone responsive element and initiates transcription. In *Atrx* Foxg1cre mice there is an increase in TBG and a decrease in T4 in the serum. In the liver, there is a decrease in *Dio1* and an increase in *Dio3*. Any T4 present is likely converted to rT3. Low levels of *Thrβ* acts as a transcriptional repressor. Following treatment with T4 in *Atrx* Foxg1cre mice, there are control levels of TBG and T4 in the blood. In the liver, *Dio1* is at control levels and *Dio3* is repressed. T4 is converted to T3 where it binds its receptor and transcription occurs normally. This occurs for a subset of genes (*Dio1, Dio3* and *Thrβ*). However, some genes (*Igf1*, *Prlr*, *Ghr* and *Thrsp*) are still transcriptionally repressed following T4 treatment. This suggests that ATRX is required for the transcription of some thyroid hormone responsive genes.

Several thyroid hormone responsive genes (*Igf1*, *Prlr*, *Ghr* and *Thrsp*) failed to respond to T4 treatment in *Atrx* Foxg1cre mice, suggesting that their induction by T4 might require ATRX. To test this, we compared the effect of T4 treatment of control and *Atrx* FoxG1cre mice on the expression of *Igf1*, *Prlr*, *Ghr* and *Thrsp* in the liver. The results show that these thyroid hormone responsive genes are induced by T4 in control mice, but not in *Atrx* mutants (Figure 6), confirming a dependence on ATRX (Figure 7).

## DISCUSSION

Low serum IGF-1 has been implicated as a driver of premature aging syndromes [19,30]. More recently, thyroid hormone signalling suppression has been linked to both premature aging and normal aging in mice [30,33]. Given the previous observation that the *Atrx* Foxg1cre mice have both low T4 and IGF1 [10], we tested the effects of T4 injections. Despite recovery of normal T4 levels, the signalling that promotes *Igf1* expression in the liver defective, likely due to previously unrecognized Cre-mediated inactivation of the *Atrx* gene in the liver. Thus, while several thyroid hormone target genes were responsive to T4 treatment, several others, including *Igf1*, were not, suggesting that ATRX is required for the induction of these genes in the liver. As a result, T4 administration does not rescue decreased lifespan, reduced growth or other defects in *Atrx* Foxg1cre mice.

Higher doses of T4 that we initially tested (0.5 and 1.0 mg/kg daily) were likely inducing hyperthyroidism in these small, young mice. The lowest dose, 0.1 mg/kg T4 daily, appeared to have variable effects as some *Atrx* cKO mice injected received well above control levels or levels similar to uninjected *Atrx* FoxG1cre mice. Whether this was experimental error or *Atrx* FoxG1cre mice have variable absorption is unknown. Nevertheless, we used only *Atrx* FoxG1cre mice that expressed control levels of serum T4 after treatment for subsequent analyses. Our results suggest that restoring normal levels of serum T4 in *Atrx* FoxG1cre mice does not ameliorate premature aging phenotypes. It is known that hyperthyroidism causes increased metabolic rate, reduced bone density and osteoporosis [34]. Due to the small size of the *Atrx* FoxG1cre mice (46.3% of control weight), it is possible that *Atrx* FoxG1cre mice received levels of T4 that are too high for their body weight and that a lower dose is necessary to eliminate induction of hyperthyroidism in this model. Since *Igf1r* appears to be unaffected by deletion of ATRX, a future experiment would be to directly inject IGF-1 in *Atrx* Foxg1cre mice. It has been shown in a Hutchinson-Gilford Progeria mouse model that IGF-1 treatment extends lifespan and delays onset of premature aging [19].

The inefficacy of T4 treatment in our study could be explained by deletion of *Atrx* in the liver of *Atrx* Foxg1cre mice, which was not previously reported. *Dio1*, *Serpina7* and *Thrβ* transcript levels were normalized by T4 treatment, indicating that hepatic T3 is produced and that thyroid signalling is at least partially intact. Conversely, several thyroid hormone responsive genes including *Igf1*, *Prlr*, *Ghr* and *Thrsp* were not rescued following T4 treatment. Furthermore, when controls were treated with T4, these genes all increased compared to control levels. These data suggest that ATRX must be required for T3-mediated transcription of a subset of genes in the liver.

It is still unknown why deletion of *Atrx* in the liver disrupts proper signalling and production of IGF-1. ATRX contains several LXXLL-type motifs required to bind hormone receptors and in the testes, it was shown that ATRX is able to bind the androgen receptor, facilitating transcription of androgen receptor target genes [15]. Therefore, it is possible that ATRX binds and cooperates with the thyroid hormone receptor directly to modulate gene expression in the liver. Moreover, a recent report strongly implicates ATRX in the regulation of gene expression in the liver [35]. It was shown that the nucleotide excision repair (NER) structure-specific endonuclease ERCC1–XPF, a complex that is mutated in premature aging, recruits the CTCF–cohesin complex, MBD2 and ATRX to promoters and imprinting control regions (ICRs) to silence a subset of imprinted genes during hepatic development [35].

ATRX deletion has been shown to result in DNA replication stress and telomere abnormalities [10]. It is also possible that in addition to low levels of IGF-1, DNA damage is accumulating in hepatocytes lacking ATRX and these cells are dying or becoming senescent, further exacerbating low levels of stem cells in the liver. In the future, it will be important to investigate the full extent of ATRX function in the liver and to further explore the link between ATRX and thyroid hormone mediated transcription.

## METHODS

### Mouse husbandry and genotyping

Mice were exposed to 12-hour light/12-hour dark cycles and fed tap water and regular chow ad libitum. The *Atrx^loxP^* mice have been described previously [36,37]. *Atrx^loxP^* mice, when mated to mice expressing Cre recombinase under the control of the *Foxg1* promoter (*Foxg1KiCre,* RRID:MGI:3767191) [38], produce male progeny with *Atrx* deficiency in the forebrain and anterior pituitary (*Atrx^loxP^;Foxg1KiCre* or *Atrx Foxg1cre* for simplicity, RRID:MGI:3530074). Ear or tail genomic DNA was used for genotyping. *Atrx*, *Cre*, and *Sry* genotyping was performed by PCR as previously described [36].

### Thyroxine injections

Control and *Atrx* Foxg1cre mice were injected subcutaneously from birth (P0) until P14 with 0.1, 0.5 or 1.0 mg/kg L-thyroxine (T4) (Sigma, T2376-100MG).

### Measurements of T4, T3, IGF-1, and glucose

Plasma samples were collected from P14 mice. Blood was collected from the inferior vena cava. EDTA pH 7.0 was added to the blood sample and centrifuged at 10000 RPM for 10 minutes at 4°C. Plasma supernatant was collected and kept frozen at –80°C. Plasma T4 was assayed using a mouse T4 ELISA kit (Calbiotech, T4044T-100) according to the manufacturers’ instructions. Plasma T3 was assayed using a mouse T3 ELISA kit (T3043T-100) according to the manufacturers’ instructions. Plasma IGF-1 content was measured using a mouse IGF-1 ELISA kit (R&D Systems, MG100) according to the manufacturers’ instructions. Blood glucose levels were measured prior to sacrifice using the ReliOn Prime Blood Glucose Meter according to the manufacturer’s instructions.

### Hematoxylin and eosin staining of skin sections

P20 mice were perfused with 4% paraformaldehyde before PBS washes and dehydration. Skin tissue was flash-frozen in liquid nitrogen using Cryomatrix (ThermoFisher Scientific) cryoprotectant [39] and sectioned at 8µm (Leica CM 3050S). H&E staining was performed as follows: CAT hematoxylin (Biocare Medical) for 2 minutes, wash for 30 seconds, Tasha’s bluing solution (Biocare Medical) for 30 seconds, wash for 10 minutes, eosin Y (Biocare Medical) for 2 minutes, 70% ethanol for 1 minute, 90% ethanol for 1 min, 100% ethanol for 4 min, Xylene for 15 minutes. Slides were mounted with permount (Fisher Scientific).

### Immunofluorescence and antibodies

P14 or P20 mice were perfused with 4% paraformaldehyde before PBS washes and dehydration. Liver tissue was flash-frozen in liquid nitrogen using Cryomatrix (ThermoFisher Scientific) cryoprotectant [39] and sectioned and sectioned at 8µm (Leica CM 3050S). For immunofluorescence staining of cryosections, antigen retrieval was performed on slides by warming 10 mM sodium citrate pH 6 solution to approximately 95°C and microwaving slides in solution for 10 minutes on low. After cooling, slides were washed, blocked with 10% normal goat serum (Sigma-Aldrich) for 1 hour and incubated with primary antibody overnight at 4°C. Slides were washed in PBS and incubated with secondary antibody for 1 hour. Sections were counterstained with DAPI (Sigma-Aldrich) and mounted with permafluor (ThermoFisher Scientific). The following primary antibodies were used: anti-ATRX, rabbit polyclonal (1:75, Santa-Cruz Biotechnology, sc-15408, RRID:AB_2061023), anti-GFP, chicken polyclonal (1:150, ThermoFisher Scientific, PA1-9533, RRID:AB_1074893), anti-RFP, rabbit polyclonal (1:150, Rockland, 600-401-379, RRID:AB_2209751), anti-Albumin, goat polyclonal (1:600, Bethyl, A90-134, RRID:AB_67120), anti-F4/80, rat monoclonal (Abcam, ab6640, RRID:AB_1140040), anti-GFAP, mouse monoclonal (Sigma-Aldrich Cat# G3893, RRID:AB_477010). The secondary antibodies used were goat anti-rabbit-Alexa Fluor 594 (1:800 dilution; ThermoFisher Scientific, A-11012, RRID:AB_2534079), goat anti-chicken-Alexa Fluor 488 (1:800 dilution; ThermoFisher Scientific, A-11039, RRID:AB_2534096), goat anti-mouse-Alexa Fluor 488 (1:800 dilution; ThermoFisher Scientific, A-21121, RRID:AB_2535764) and goat anti-rat-Alexa Fluor 488 (1:800 dilution; Thermo Fisher Scientific, A-11006, RRID:AB_2534074).

### Microscopy and imaging

Hematoxalin and eosin images were captured using a scanscope (Aperio CS model, Leica). Scanscope console imaging software was used for automated image capture and processing was performed using Volocity software (PerkinElmer). Immunofluorescence images were captured using an inverted microscope (DMI 6000b, Leica). Digital microscopy images were captured with a digital camera (ORCA-ER, Hamamatsu). Openlab imaging software was used for manual image capture, and processing was performed using Volocity software (PerkinElmer).

### Quantitative reverse transcriptase PCR (qRT-PCR)

Total RNA was isolated from liver with the RNeasy Mini Kit (QIAGEN) and reverse transcribed into cDNA as described previously [40]. Control reactions without reverse transcriptase were prepared in parallel. cDNA was amplified with gene-specific primers under the following conditions: 25–35 cycles of 95°C for 30 seconds, 60°C for 30 seconds, and 72°C for 1 minute. For qRT-PCR, cDNA was amplified with iQ SYBR Green Master Mix (Bio-Rad) by using the standard curve Ct method of quantification. Experiments were performed on a Chromo-4 thermocycler (MJ Research) and analyzed with Opticon Monitor 3 and GeneX (Bio-Rad) software. Gene expression analysis was repeated in duplicate for each sample. Conditions for amplification were as follows: 35 cycles of 95°C for 10 seconds, 55°C for 20 seconds, 72°C for 30 seconds, and a final melting curve generated in increments of 0.5°C per plate read. Primer sequences are listed in Table 1.

**Table 1:** Primer set sequences.

Gene Name	Forward Primer	Reverse Primer
*Igf1*	Acc tca gac agg cat tgt gg	gtt tgt cga tag gga cgg gg
*Igf1r*	tgt ggt caa gga tga acc cg	cct tgg gat acc aca ccc ag
*Igf2r*	gca tct ttc cac cag ttc cg	gct cgt cct cat tgt cat cc
*Igfals*	cac aca acg cca tca cta gc	cgt tga aga ggc caa aga ag
*Igfbp1*	agc cca gag atg aca gag ga	gtt ggg ctg cag cta atc tc
*Igfbp2*	gcg ggt acc tgt gaa aag ag	aac aca gcc agc tcc ttc at
*Igfbp3*	gtg acc gat tcc aag ttc ca	tgt cct cca ttt ctc tgc gg
*Serpina7*	cct tcc aaa aga ggg aca ca	cca agg tca tat gtg gca ga
*Dio1*	gga cac aat gca gaa cca ga	gca aag ctt ttc cag gac ag
*Dio3*	gtt ttt ggc ttg ctc tca gg	caa caa gtc cga gct gtg aa
*Thrβ*	cag aac cca cgg atg agg ag	ggc att cac aat ggg tgc tt
*Prlr*	gca tct ttc cag cag ttc cg	gct cgt cct cat tgt cat cc
*Ghr*	att cac caa gtg tcg ttc c	tcc att cct ggg tcc att ca
*Thrsp*	acg gag ccc ctg atc tct at	ggc ttc tag gtc cag ctc ct

## Statistical analysis

Statistical analysis was performed using GraphPad Prism6 software (6.05; GraphPad Software Inc.), and all results are expressed as the mean ± SEM unless indicated otherwise. Two independent data sets were compared with the Student’s t test (unpaired, 2-tailed). Multiple independent data sets were compared with a one-way ANOVA with post-hoc Tukey’s test. Statistical analyses of Kaplan-Meier survival curves were performed using the log-rank test and the Gehan-Breslow-Wilcoxon test. A repeated-measures polynomial modeling analysis (SAS v.9.4, SAS Institute Inc., Cary, NC, USA) was used to compare differences between groups in weight over time. P values of 0.05 or less were considered to indicate significance.

### Study approval

All procedures involving animals were conducted in accordance with the regulations of the Animals for Research Act of the Province of Ontario and approved by the University of Western Ontario Animal Care and Use Committee.

## Supplementary Material

Supplementary File
