# Supplementary material for: Inactivation of hepatic ATRX in Atrx Foxg1cre mice prevents reversal of aging-like phenotypes by thyroxine
Source: Aging (Albany NY). 2018 Jun 7;10(6):1223–38. doi: 10.18632/aging.101462 (PMC6046231; doi:10.18632/aging.101462)
Supplement: Supplementary File [file aging-10-101462-s001.pdf]

SUPPLEMENTARY MATERIAL

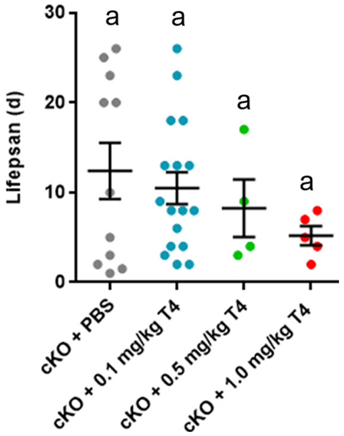

Supplementary Figure 1. T4 supplementation does not improve lifespan in *Atrx* FoxG1cre (cKO) mice. Average lifespan was not significantly changed between PBS and T4 treated *Atrx* FoxG1cre mice. Groups with the same letter have means that are not significantly different. Groups with different letters have means that are significantly different ($p<0.05$). Error bars represent SEM.

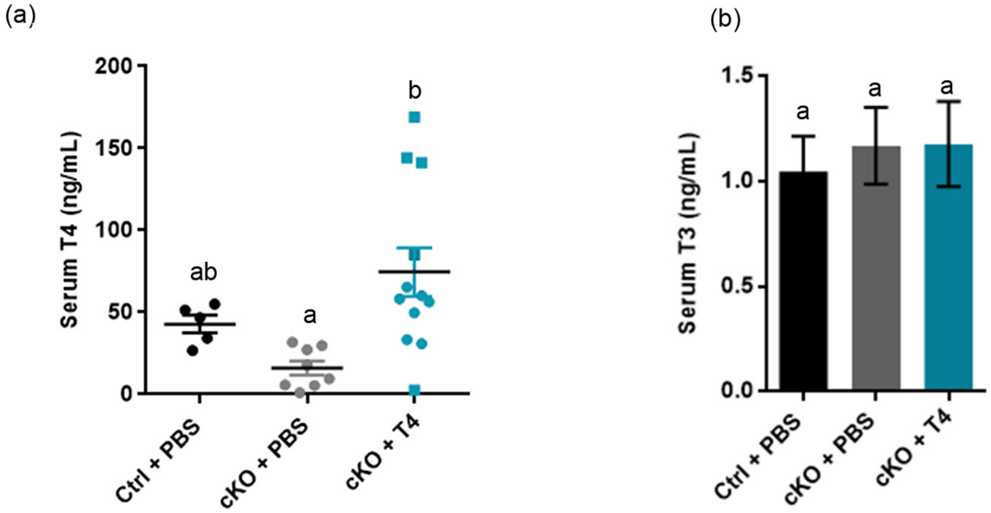

Supplementary Figure 2. Variable serum T4 levels and unchanged serum T3 levels in *Atrx* FoxG1cre (cKO) mice upon T4 supplementation. (**a**) Graph of Serum T4 levels in *Atrx* FoxG1cre mice following T4 treatment. Biological outliers are represented as squares and these mice were excluded from further analyses. (**b**) Graph of serum T3 levels in *Atrx* FoxG1cre mice following T4 treatment. Groups with the same letter have means that are not significantly different. Groups with different letters have means that are significantly different ($p<0.05$). Error bars represent SEM.

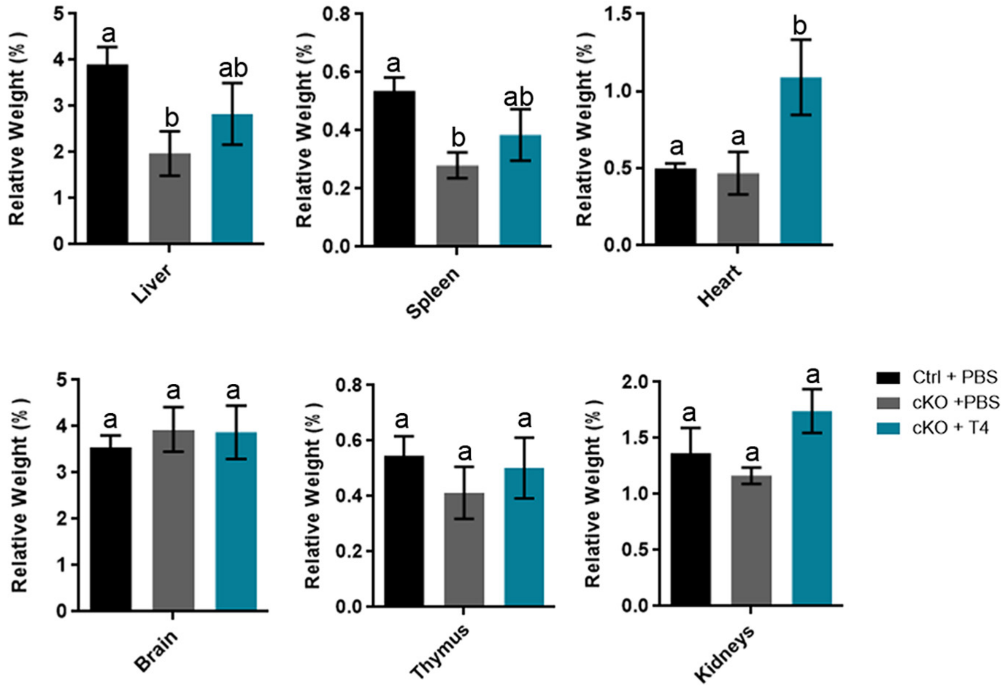

Supplementary Figure 3. Liver and spleen abnormalities in *Atrx* FoxG1cre (cKO) mice are partially rescued following T4 treatment. Following T4 treatment in *Atrx* FoxG1cre mice, abnormal relative organ weights in the liver and spleen are statistically similar to PBS treated control. There is an increase in relative heart weight in T4 treated *Atrx* FoxG1cre mice compared to PBS treated control mice. There was no significant difference in brain, thymus or kidneys relative weight. Groups with the same letter have means that are not significantly different. Groups with different letters have means that are significantly different (p<0.05). Data were normalized to body weight, n=3-6. Error bars represent SEM.
